# Attitudes towards healthy eating and its determinants among older adults in a deprived region of Hungary: implications for the National Healthy Aging Program

**DOI:** 10.1007/s11357-025-01533-9

**Published:** 2025-01-28

**Authors:** Nora Kovacs, Eva Biro, Peter Piko, Zoltan Ungvari, Roza Adany

**Affiliations:** 1 Department of Public Health and Epidemiology, Faculty of Medicine, University of Debrecen, Debrecen, Hungary; 2 HUN-REN–UD Public Health Research Group, Department of Public Health and Epidemiology, Faculty of Medicine, University of Debrecen, Debrecen, Hungary; 3 National Laboratory for Health Security, Center for Epidemiology and Surveillance, Semmelweis University, Budapest, Hungary; 4 International Training Program in Geroscience, Doctoral College, Health Sciences Program/Institute of Preventive Medicine and Public Health, Semmelweis University, Budapest, Hungary; 5 Vascular Cognitive Impairment and Neurodegeneration Program, Oklahoma Center for Geroscience and Healthy Brain Aging, Department of Biochemistry and Molecular Biology, University of Oklahoma Health Sciences Center, Oklahoma City, OK USA; 6 Department of Health Promotion Sciences, College of Public Health, University of Oklahoma Health Sciences Center, Oklahoma City, OK USA; 7 Institute of Preventive Medicine and Public Health, Semmelweis University, Budapest, Hungary

**Keywords:** Healthy eating, Attitude, Barriers, Mental health, Ageing

## Abstract

A healthy diet is a key determinant of successful aging. However, the psychological, social, and physiological changes associated with ageing often disrupt dietary behaviours. Hungary has one of the highest rates of chronic age-related diseases in the European Union, exacerbated by unhealthy dietary patterns and rapid population aging. This study evaluates attitudes and barriers to healthy eating among older adults in a socioeconomically disadvantaged region of Hungary, identifying determinants of these attitudes across different age groups. A cross-sectional survey sampled 678 adults aged 18 and older from Northeast Hungary, assessing their beliefs on healthy eating through an attitude score (range: 9–45). Multivariate regression analyses were conducted to explore relationships between mental health, self-perceived health, and attitudes toward healthy eating. The mean attitude score was 31.47 (± 5.14), with older adults (≥ 65 years) showing significantly greater uncertainty about dietary choices. The cost of healthy food was the most frequently reported barrier, regardless of age. Regression results revealed that older adults with higher well-being (B = 0.03, p = 0.026), life satisfaction (B = 0.40, p = 0.005), and self-perceived health (fair: B = 2.20, p = 0.003; good/very good: B = 1.96, p = 0.031) were more likely to have positive attitudes toward healthy eating. These results emphasize the importance of addressing both mental and physical health in educational interventions to promote healthier diets. Tailored approaches addressing affordability and accessibility of healthy foods are critical to advancing Hungary’s National Healthy Aging Program and mitigating dietary risk factors among vulnerable populations.

## Introduction

The rapid aging of the global population presents a significant public health challenge. By 2050, 16% of the world’s population is expected to be aged 65 years or older, highlighting the pressing need for tailored health policies and interventions [1]. In the European Union (EU), aging trends are even more pronounced. Projections indicate that those aged 65 and over will comprise 29% of the EU population by 2050, from 21.3% in 2023, while the population aged 85 and above is anticipated to more than double, from 12.5 million in 2019 to 26.8 million by 2050 [2, 3]. Hungary reflects these trends, with its proportion of people aged 65 and older rising from 20.6% in 2022 to a projected 26.9% by 2050, underscoring the urgency of addressing the unique health challenges posed by an aging population [3].

As life expectancy increases, chronic non-communicable diseases (NCDs) remain the leading causes of morbidity and disability among older adults [4]. Unhealthy diet represents a significant modifiable risk factor for numerous chronic conditions, including cardiovascular diseases, type 2 diabetes mellitus, cancer, and obesity-related disorders [5]. In Hungary, dietary risks contributed to 25% of all deaths in 2019, far exceeding the EU average of 17%, and obesity prevalence ranks as the second highest among EU member states [6]. These alarming statistics call for urgent action to reduce dietary risks, improve nutritional habits, and reduce the burden of age-related chronic diseases. Diet is a key determinant of successful aging, promoting health, well-being, and quality of life. However, aging-related psychological, social, and physiological changes often disrupt eating behaviours, creating barriers to adopting healthy dietary patterns [7, 8]. Psychosocial factors, such as mental well-being and social support, can either facilitate or hinder healthy eating in later life, and their interaction with eating behaviours may be bidirectional [9]. Moreover, practical challenges such as income constraints, living conditions, functional difficulties, and limited access to nutritious foods have a particular impact on socioeconomically disadvantaged populations [10]. Older adults who are food insecure are more likely to have lower intakes of energy and essential nutrients, to be in poor health, and to experience limitations in daily activities [10]. Addressing these barriers through targeted strategies is critical to promoting dietary change and improving health outcomes in older adults.

Encouraging older individuals to adopt healthier dietary habits has been shown to significantly improve quality of life and reduce the burden of chronic diseases [11, 12]. Previous studies demonstrated that greater knowledge and positive attitudes towards healthy eating are strongly associated with positive dietary behaviours, such as increased consumption of fruits and vegetables and active information-seeking about nutrition [13]. Furthermore, nutrition-related knowledge and attitudes positively influence the health status and quality of life among the older population by promoting behavior change [14].

Since attitudes are key predictors of future behavior [15], gaining a deeper understanding of older adults’ perceptions of and barriers to healthy eating is crucial for developing effective interventions. Identifying the factors that affect these attitudes and encourage engagement in healthy behaviors has significant implications for public health strategies aimed at promoting healthy ageing. This is particularly important in Hungary, where existing national surveys revealed unfavorable dietary patterns characterized by high consumption of fats, cholesterol, salt, and sugar, and inadequate consumption of fruits and vegetables [16, 17].

Although older people may be at greater risk of both mental health problems [18] and unhealthy eating attitudes [7], there is limited research examining the interplay of these issues among socioeconomically disadvantaged populations. This study aims to address this research gap by investigating the attitudes and barriers to healthy eating among older adults in Northeast Hungary, one of the EU’s most deprived areas [19]. Specifically, we aim to explore how mental health, self-perceived health, and socioeconomic factors influence these attitudes across age groups. The findings from this research are expected to serve as an essential basis for Hungary’s National Healthy Aging Program.

## Methods

### Study design and population

The present study utilised data from two cross-sectional surveys conducted in 2018 and 2022, respectively. Participants were recruited from two counties (Hajdú-Bihar and Szabolcs-Szatmár-Bereg counties) in North-East Hungary. The first survey (2018) involved adults aged 18–64 years, who were randomly selected from the patients’ lists of 20 general practitioners (GPs). The second survey, performed in 2022, involved randomly selected individuals aged 65 years and older who were registered by 19 GPs from the same region. From each GP practice, 25 individuals were invited to participate in the surveys. Altogether, the study sample comprised 860 individuals, including 417 participants recruited in the first survey and 443 in the second survey.

The two surveys consisted of three main components (questionnaire-based, physical, and laboratory investigations) allowing a comprehensive health assessment. Details of the study design and data collection process for the surveys have been described elsewhere [20, 21].

The research was conducted in accordance with the Declaration of Helsinki, and the protocols were approved by the Ethics Committee of the Hungarian Scientific Council on Health (Reference No.: 61327–2017/EKU and IV/3351–3/2022/EKU). We obtained written informed consent from all participants involved in the surveys.

### Identification of attitudes and barriers to healthy eating

The survey included a healthy eating-related attitude questionnaire that was used in the Food and You survey (Wave 4) [22, 23]. The original English version was translated into Hungarian and the accuracy of the translation was checked by back-translating into English by an independent translator blinded to the original questionnaire. These versions were reviewed by the research team, and the final Hungarian version was developed based on the consensus of the team members, including a nutrition specialist. In the next stage, a cognitive test and a pilot test were carried out prior to its use in our study. Participants were asked to express their attitudes and beliefs about various aspects of healthy eating with a range of statements. The section consisted of 9 questions in relation to their views on importance of and motivations for adopting a healthy diet. A 5-point Likert scale ranging from 1 (completely agree) to 5 (completely disagree) was used to assess their attitudes. Reverse coding was used for two items (questions 4 and 9) to calculate the total score. The total attitude score, ranging from 9 to 45, was calculated by summing the raw scores for the nine questions, with higher scores reflecting more positive attitudes towards healthy eating. After excluding individuals with incomplete data for the questions on healthy eating attitudes, the final sample included 678 individuals (*n* = 327 and *n* = 351 for 18–64 years and 65 years and older, respectively).

Participants were also asked to choose one or more of the 4 diets listed to indicate what they considered to be a healthy diet. Participants were also asked to select one or more of the 6 response options to identify the difficulties they have in following a healthy diet. This question was developed based on the open-ended question used in the Food and You Survey [23].

### Sociodemographic and health-related variables covered

The questionnaires covered demographic and socioeconomic data including age, sex, education level and self-reported financial status. Education level was classified into primary or less, secondary, and tertiary. Self-reported financial status was divided into bad/very bad, satisfactory or very good/good. In addition to demographic and socioeconomic data, health-related data were also collected including physical and mental health. Body mass index (BMI) was calculated as body weight in kilogrammes divided by height in metres squared (kg/m^2^) and was classified as underweight/normal (< 25.0 kg/m^2^), overweight (BMI: 25.0–29.9 kg/m^2^) and obese (BMI: ≥ 30.0 kg/m^2^). Self-rated health was assessed using five categories ranging from very bad to very good which were collapsed into three categories for analysis: very bad/bad, fair and good/very good. Mental health was evaluated using three measures: well-being, life satisfaction and psychological distress [18].

The 5-item World Health Organization Well-Being Index (WHO-5) was used to evaluate the well-being of the study participants [24]. The self-report measure, which was developed by the World Health Organization, employs a 6-point Likert scale ranging from 0 (not at all) to 5 (all the time) to assess the mental well-being over the past two weeks. The raw score was multiplied by 4 to obtain the final score (ranging from 0 to 100), with higher scores indicating better mental well-being. A score < 50 indicates poor emotional well-being and suggests a need for further investigation [24].

Life satisfaction was measured by a single-item measure, where participants were asked to rate their overall life satisfaction on a scale ranging from 0 (completely dissatisfied) to 10 (completely satisfied). A higher value represented greater life satisfaction. Previous research has demonstrated the reliability and validity of single-item measures of life satisfaction, with results comparable to those of multi-item measures [25, 26].

The twelve-item General Health Questionnaire (GHQ-12) was used to assess psychological distress [27]. The Likert scoring method was used, where the total score (ranging from 0 to 36) was calculated as the sum of each item scored on a four-point Likert scale (0–1–2–3), with higher scores indicating an increased likelihood of psychological distress.

### Statistical analysis

Descriptive statistics were used to describe the characteristics of the study population. Data were presented as mean and standard deviation (SD) for continuous variables and proportions (%) for categorical variables. Mann–Whitney U test and Kruskal–Wallis test were used for non-normally distributed continuous variables. Response options on the 5-point Likert scale regarding healthy eating attitudes were collapsed into three categories: strongly agree/agree categories, uncertain and strongly disagree/disagree. Differences between groups of categorical variables were tested using Pearson’s chi-squared test.

Multiple linear regression analysis was applied to investigate the association between sociodemographic variables, health-related measures and the attitude scores. Models 1–4 have been developed to examine these associations by including a set of baseline variables across all models. To assess the specific contributions of mental and physical health measures, the baseline model was extended by adding the specific health measure (well-being, life satisfaction, psychological distress and self-rated health) to each model. The analysis was stratified by age group (18–64 years and 65 years and over) to allowing comparative analysis of patterns of healthy eating attitudes across age groups. Bootstrapping with 1,000 resamples was applied in the regression analyses. Results were presented as regression coefficients (B), odds ratios (OR) and corresponding 95% confidence intervals (CI). A *p* < 0.05 was considered statistically significant. Stata version 13.0 software (Stata Corp., College Station, TX, USA) was used for statistical analyses.

## Results

Table 1 presents the sociodemographic characteristics of the study participants by age group. The mean age of the participants (*n* = 678) was 58.34 (± 17.62) years. Those aged 65 years and older were more likely to have lower levels of education (*p* < 0.001), to report worse financial status (p = 0.006) and to have significantly higher BMI (*p* < 0.001). Among older adults, less than a quarter (23.85%) rated their health as very good or good compared to 66.75% of younger participants (*p* < 0.001). A significantly worse mental health was reported by older people compared to those aged 18–64 in terms of life satisfaction (7.22 (± 2.03) vs. 7.78 (± 1.78), *p* < 0.001) and psychological distress (10.33 (± 5.39) vs. 9.13 (± 4.7), *p* < 0.001). However, there was no significant difference in well-being between age groups (p = 0.079).

**Table 1:** Characteristics of the study population by age group

			Age groups		
Characteristics		Total (*n* = 678)	18–64 years (*n* = 327)	≥ 65 years (*n* = 351)	*p*-value
		n (%)	n (%)	n (%)	
Sex	male	275 (40.56%)	142 (43.43%)	133 (37.89%)	0.143
	female	403 (59.44%)	185 (56.57%)	218 (62.11%)	
Age	mean (SD)	58.34 (17.62)	43.20 (12.75)	72.45 (5.94)	
Education level	primary	191 (28.3%)	65 (20%)	126 (36%)	< 0.001*
	secondary	376 (55.7%)	198 (60.92%)	178 (50.86%)	
	tertiary	108 (16%)	62 (19.08%)	46 (13.14%)	
Self-reported financial status	very good/good	192 (28.83%)	111 (34.58%)	81 (23.48%)	0.006*
	satisfactory	399 (59.91%)	178 (55.45%)	221 (64.06%)	
	bad/very bad	75 (11.26%)	32 (9.97%)	43 (12.46%)	
BMI	underweight/normal	191 (28.17%)	116 (35.47%)	75 (21.37%)	< 0.001*
	overweight	254 (37.46%)	113 (34.56%)	141 (40.17%)	
	obese	233 (34.37%)	98 (29.97%)	135 (38.46%)	
Self-perceived health	very bad/bad	65 (9.63%)	11 (3.36%)	54 (15.52%)	< 0.001*
	fair	309 (45.78%)	98 (29.97%)	211 (60.63%)	
	good/very good	301 (44.59%)	218 (66.67%)	83 (23.85%)	
Well-being index (WHO-5)	mean (SD)	69.69 (18.07)	71.24 (16.93)	68.25 (18.98)	0.079
Psychological distress (GHQ-12)	mean (SD)	9.75 (5.10)	9.13 (4.7)	10.33 (5.39)	< 0.001*
Single-item Life Satisfaction Scale (LS)	mean (SD)	7.49 (1.93)	7.78 (1.78)	7.22 (2.03)	< 0.001*
Healthy eating attitude score	mean (SD)	31.47 (5.14)	31.82 (5.18)	31.15 (5.09)	0.158

*p < 0.05 statistically significant. SD, standard deviation

The mean healthy eating attitude score was 31.47 (± 5.14). Although no significant differences were found in the healthy eating attitude score between the two age-groups (p = 0.158), the average score was slightly higher (31.82 ± 5.18) for the 18–64 years age group than for the 65 years or older age group (31.15 ± 5.09).

The majority of respondents, regardless of age, agreed that a balanced diet rich in fruits and vegetables could be considered a healthy diet. However, those under aged 65 were more than twice as likely to consider a high-calorie diet to be healthy (*p* < 0.001) (Table 2).

**Table 2:** Healthy eating

	Age groups				
	18–64 (*n* = 402)		65 + (*n* = 416)		*p*-value
	n	%	n	%	
High-calorie diet	57	14.2%	27	6.5%	< 0.001*
Vegetarian diet	5	1.2%	3	0.7%	0.448
Balanced diet rich in vegetables and fruits	323	80.3%	353	84.9%	0.089
Low fat diet	58	14.4%	77	18.5%	0.116

*p < 0.05 statistically significant

A quarter of the respondents (27.8% in the 18–64 age group and 24.7% in the 65 years and older) reported having no barriers to follow a healthy diet. The most frequently mentioned barrier was the cost of healthy foods, followed by the time to prepare healthy food in both age groups, but the proportion of those who marked the cost was significantly higher among the elderly. Significant differences were found across educational levels regarding the proportion of respondents reporting no barriers and cost of healthy foods in both age groups (*p* < 0.001). In addition, older adults with a lower education level were more likely to report the time of cooking (p = 0.040) and access to healthy foods (p = 0.033) as barriers compared to those with higher level of education. (Table 3).

**Table 3:** Difficulties in trying to eat healthily by educational level

	18–64 years					65 years and older					p-value
	total	primary	secondary	tertiary	p-value	total	primary	secondary	tertiary	p-value	
	n (%)	n (%)	n (%)	n (%)		n (%)	n (%)	n (%)	n (%)		
No difficulties / already eat healthily	116 (27.8%)	15 (16.9%)	65 (25.9%)	35 (46.7%)	** < 0.001**	103 (24.7%)	17 (10.9%)	57 (27.9%)	29 (51.8%)	** < 0.001**	0.297
Healthy foods are too expensive	222 (53.2%)	60 (67.4%)	137 (54.6%)	24 (32%)	** < 0.001**	274 (65.7%)	127 (81.4%)	124 (60.8%)	22 (39.3%)	** < 0.001**	** < 0.001**
Time to prepare / cook healthy food	72 (17.3%)	15 (16.9%)	42 (16.7%)	15 (20%)	0.799	77 (18.5%)	37 (23.7%)	35 (17.2%)	5 (8.9%)	**0.040**	0.663
Don’t know which food is healthy	27 (6.5%)	9 (10.1%)	15 (6%)	2 (2.7%)	0.140	30 (7.2%)	14 (9%)	13 (6.4%)	2 (3.6%)	0.354	0.687
Don’t like healthy food	18 (4.3%)	5 (5.6%)	11 (4.4%)	2 (2.7%)	0.651	18 (4.3%)	7 (4.5%)	10 (4.9%)	1 (1.8%)	0.593	0.994
Can't get healthy food in the store where I usually shop	32 (7.7%)	7 (7.9%)	18 (7.2%)	7 (9.3%)	0.826	34 (8.2%)	19 (12.2%)	14 (6.9%)	1 (1.8%)	**0.033**	0.805
Other	14 (3.4%)	0 (0%)	10 (4%)	4 (5.3%)	0.118	13 (3.1%)	5 (3.2%)	8 (3.9%)	0 (0%)	0.327	0.840

Statistically significant results (*p* < 0.05) are shown in bold

Significant differences were found in healthy eating attitude among age groups. More participants aged 18–64 in comparison to the older participants (aged 65 years and older) agreed the statement that the main reason for people to eat a more healthy diet is to lose weight (43.4% vs. 29.6%; *p* < 0.001), and that the experts contradict each other over what foods are good or bad for them (47.7% vs. 36.8%; p = 0.015). Nevertheless, participants from the 18–64 years age group were less likely to confirm the statements that good health is just a matter of good luck (13.1% vs. 20.2%; p = 0.037) and you can eat what you like if you are not overweight (20.8% vs. 28.8%, p = 0.046) compared with those aged 65 years and older. In general, older participants showed a significantly higher level of uncertainty regarding healthy eating. More older participants (aged 65 years and older) in comparison to the 18–64 years age group expressed their uncertainty about the statements that 1) the tastiest foods are the ones that are bad for them, 2) as long as they take enough exercise they can eat whatever they want, 3) the main reason for people to eat a more healthy diet is to lose weight, and 4) that the experts contradict each other over what foods are good or bad for them (see Table 4).

**Table 4:** Attitudes towards healthy eating

	Age groups												
	18–64						65 +						
	Strongly agree/agree		Uncertain		Strongly disagree/disagree		Strongly agree/agree		Uncertain		Strongly disagree/disagree		p-value
	n	%	n	%	n	%	n	%	n	%	n	%	
The tastiest foods are the ones that are bad for you	64	19.6%	66	20.2%	197	60.2%	66	18.8%	101	28.8%	184	52.4%	0.031*
I get confused over what’s supposed to be healthy and what isn’t	143	43.7%	92	28.1%	92	28.1%	122	34.8%	115	32.8%	114	32.5%	0.057
If you are not overweight you can eat what you like	68	20.8%	54	16.5%	205	62.7%	101	28.8%	58	16.5%	192	54.7%	0.046*
Small dietary changes, such as eating less fat or cutting down on sugar, can lead to benefits for my future health	258	78.9%	39	11.9%	30	9.2%	271	77.2%	52	14.8%	28	8.0%	0.497
As long as you take enough exercise you can eat whatever you want	83	25.4%	64	19.6%	180	55.0%	87	24.8%	105	29.9%	159	45.3%	0.005*
The main reason for people to eat a more healthy diet is to lose weight	142	43.4%	68	20.8%	117	35.8%	104	29.6%	129	36.8%	118	33.6%	< 0.001*
Good health is just a matter of good luck	43	13.1%	55	16.8%	229	70.0%	71	20.2%	61	17.4%	219	62.4%	0.037*
The experts contradict each other over what foods are good or bad for you	156	47.7%	84	25.7%	87	26.6%	129	36.8%	112	31.9%	110	31.3%	0.015*
What you eat makes a big difference to how healthy you are	232	70.9%	59	18.0%	36	11.0%	244	69.5%	70	19.9%	37	10.5%	0.817

*p < 0.05 statistically significant

Multiple regression analyses showed that higher well-being was associated with better attitude in both age groups (aged 18–64 years: B = 0.05, p = 0.013; 65 years and above: B = 0.03, p = 0.026). Similarly, more positive attitude was associated with higher life satisfaction in aged 18–64 years: B = 0.69, *p* < 0.001 and aged 65 years and above: B = 0.40, p = 0.005. However, psychological distress was associated with lower attitude score only in aged 18–64 (B = −0.20, p = 0.002). Older participants who rated their health as fair (B = 2.20, p = 0.003) and good or very good (B = 1.96, p = 0.031) were more likely to report better attitude towards health eating. Higher level of education and bad/very bad self-reported financial status showed association with attitude score only in the 18–64 age group, while obesity was associated with significantly lower attitude scores only among older adults. (Tables 5 and 6).

**Table 5:** Multiple regression analysis between socioeconomic, physical and mental health and healthy eating attitude among 18–64 year olds

	18–64 years old											
	Model 1			Model 2			Model 3			Model 4		
	B	95% CI	p-value	B	95% CI	p-value	B	95% CI	p-value	B	95% CI	p-value
Sex (ref. male)												
female	0.91	(−0.23, 2.05)	0.118	1.00	(−0.09, 2.09)	0.073	0.94	(−0.22, 2.09)	0.113	**1.17**	**(0.06, 2.29)**	**0.039**
Age	0.02	(−0.03, 0.06)	0.390	0.02	(−0.02, 0.07)	0.292	0.01	(−0.03, 0.05)	0.662	0.03	(−0.01, 0.08)	0.159
Education (ref. primary)												
secondary	0.94	(−0.64, 2.52)	0.244	0.78	(−0.78, 2.34)	0.329	0.91	(−0.68, 2.5)	0.263	0.79	(−0.8, 2.39)	0.331
tertiary	**2.46**	**(0.59, 4.34)**	**0.010**	**2.18**	**(0.39, 3.97)**	**0.017**	**2.39**	**(0.52, 4.26)**	**0.012**	**2.11**	**(0.27, 3.95)**	**0.024**
Self-reported financial status (ref. very good/good)												
satisfactory	−0.50	(−1.71, 0.72)	0.423	−0.44	(−1.59, 0.7)	0.448	−0.73	(−1.92, 0.46)	0.230	−0.52	(−1.72, 0.68)	0.394
bad/very bad	**−2.47**	**(−4.58, −0.37)**	**0.021**	**−2.25**	**(−4.44, −0.06)**	**0.044**	**−2.27**	**(−4.42, −0.13)**	**0.038**	**−2.50**	**(−4.67, −0.34)**	**0.023**
BMI (ref. underweight/normal)												
overweight	0.65	(−0.74, 2.05)	0.361	0.45	(−0.88, 1.77)	0.509	0.6	(−0.76, 1.96)	0.386	0.43	(−0.94, 1.79)	0.540
obese	0.97	(−0.51, 2.44)	0.199	0.68	(−0.75, 2.1)	0.351	0.93	(−0.48, 2.33)	0.197	0.98	(−0.45, 2.41)	0.181
Well-being	**0.05**	**(0.01, 0.08)**	**0.013**									
Life satisfaction				**0.69**	**(0.35, 1.03)**	** < 0.001**						
Psychological distress							**−0.2**	**(−0.32, −0.07)**	**0.002**			
Self-rated health (ref. very bad/bad)												
fair										0.74	(−2.64, 4.12)	0.667
good/very good										2.86	(−0.55, 6.26)	0.100

Statistically significant results (*p* < 0.05) are shown in bold. BMI, Body mass index; B, linear regression coefficient; CI, confidence interval. All models include the same set of variables except mental and physical health measures

**Table 6:** Multiple regression analysis between socioeconomic, physical and mental health and healthy eating attitude among people aged 65 years and older

	65 years and older											
	Model 1			Model 2			Model 3			Model 4		
	B	95% CI	p-value	B	95% CI	p-value	B	95% CI	p-value	B	95% CI	p-value
Sex (ref. male)												
female	0.68	(−0.48, 1.84)	0.248	0.5	(−0.61, 1.6)	0.378	0.57	(−0.56, 1.69)	0.326	0.5	(−0.58, 1.58)	0.361
Age	−0.05	(−0.15, 0.05)	0.340	−0.05	(−0.15, 0.05)	0.342	−0.05	(−0.16, 0.05)	0.292	−0.03	(−0.13, 0.07)	0.501
Education (ref. primary)												
secondary	0.37	(−0.84, 1.57)	0.553	0.3	(−0.97, 1.58)	0.641	0.25	(−1.04, 1.53)	0.707	0.53	(−0.7, 1.76)	0.397
tertiary	0.89	(−0.99, 2.77)	0.354	0.9	(−1.05, 2.86)	0.364	0.82	(−1.09, 2.74)	0.401	0.96	(−1.03, 2.95)	0.342
Self-reported financial status (ref. very good/good)												
satisfactory	0.15	(−1.28, 1.57)	0.842	0.26	(−1.12, 1.65)	0.709	0.05	(−1.35, 1.45)	0.946	−0.13	(−1.42, 1.17)	0.849
bad/very bad	−1.84	(−3.82, 0.15)	0.070	−1.67	(−3.63, 0.29)	0.094	−1.74	(−3.71, 0.23)	0.084	**−1.98**	**(−3.8, −0.15)**	**0.034**
BMI (ref. underweight/normal)												
overweight	−1.21	(−2.56, 0.15)	0.081	−1.30	(−2.69, 0.09)	0.066	−1.10	(−2.47, 0.26)	0.112	−1.04	(−2.3, 0.22)	0.107
obese	**−1.40**	**(−2.79, −0.01)**	**0.048**	**−1.40**	**(−2.74, −0.06)**	**0.041**	−1.28	(−2.61, 0.06)	0.062	−1.22	(−2.57, 0.14)	0.079
Well-being	**0.03**	**(0.00, 0.06)**	**0.026**									
Life satisfaction				**0.40**	**(0.12, 0.67)**	**0.005**						
Psychological distress							−0.10	(−0.2, 0.01)	0.079			
Self-rated health (ref. very bad/bad)												
fair										**2.20**	**(0.76, 3.65)**	**0.003**
good/very good										**1.96**	**(0.18, 3.74)**	**0.031**

Statistically significant results (*p* < 0.05) are shown in bold. BMI, Body mass index; B, linear regression coefficient; CI, confidence interval. All models include the same set of variables except mental and physical health measures

## Discussion

This study provides valuable insights into the beliefs and attitudes regarding healthy eating among individuals living in a socioeconomically disadvantaged region of Hungary. Our findings highlight the critical role of mental and physical health in affecting healthy eating attitudes, particularly among older adults. Cost emerged as a significant barrier across all age groups, underscoring the socioeconomic challenges faced in adopting healthier diets.

Information transfer to the population is inevitable to facilitate people to make informed decisions regarding their diet and lifestyle [28]. In addition, promoting positive attitudes towards healthy eating and behaviour may be a key to improving population adherence to dietary guidelines [29]. While the majority of respondents recognized a balanced diet rich in fruits and vegetables as healthy, younger participants (18–64 years) were more likely to consider high-calorie diets as healthy. This discrepancy emphasizes the need for targeted interventions addressing misconceptions about nutrition. Although participants generally agreed that dietary patterns influence health, overall uncertainty was about what constitutes a healthy diet, particularly among older adults (≥ 65 years). This uncertainty extended to motivations for healthy eating, such as weight management, reflecting the need for clearer and more consistent messages about dietary guidelines.

Interestingly, no significant difference in mean attitude scores was observed between age groups. This may be explained by the findings of our previous study, as premature mortality in highly disadvantaged populations likely excluded the most vulnerable individuals from the study, resulting in a relatively healthier population with higher health literacy and more positive health attitudes [21]. Our results also suggest that among older adults, attitudes toward healthy eating are more strongly influenced by health status than by sociodemographic factors such as education level, financial status, or sex. In contrast, socioeconomic factors showed stronger associations with attitudes in younger adults, highlighting various determinants of dietary perceptions across life stages.

Notably, older adults with a lower body mass index (BMI) exhibited more positive attitudes toward healthy eating, consistent with previous research showing that favourable health attitudes are associated with better weight management in later life [30]. These findings suggest that health education programs should prioritize addressing health behaviours in individuals with higher BMI to encourage positive dietary changes. Our results indicate that subjective health and mental health of individuals in older age might have positive association with healthy eating attitudes. The association, however, may be bidirectional, with a more positive attitude leading to an improved health status, or conversely, an improved health status contributing to a more positive attitude [14].

Mental health problems as depressive symptoms and psychological distress have been linked to unhealthy eating behaviours, which, in turn, increase the risk of obesity and metabolic conditions [31, 32]. Furthermore, a previous study demonstrated that social ideological beliefs (including materialism that is associated with worse psychological well-being, reduced life satisfaction and depression [33]), may, in turn, have a negative impact on health attitudes, which may influence the consumption of healthy and unhealthy foods [30]. In this study, better well-being and higher life satisfaction were positively associated with healthier eating attitudes in older adults, while psychological distress affected attitudes primarily in younger participants. These results highlight the need to address mental health concerns as part of comprehensive dietary interventions, particularly for older populations [28].

The perceived high cost of healthy foods was a main barrier for participants. This is consistent with findings from previous studies that identified financial constraints as a major barrier to health-promoting behaviours in disadvantaged populations [34, 35]. Addressing these perceptions through community-based initiatives, or other cost-reduction strategies could significantly enhance dietary behaviour.

While this study provides meaningful insights, several limitations should be noted. The cross-sectional design restricts our ability to draw causal inferences, as it remains unclear whether healthier attitudes influence mental health or vice versa. Additionally, the focus on a specific disadvantaged region limits the generalizability of the findings to Hungary’s broader population. Self-reported data on socioeconomic and health status may introduce bias, and the timing of the second survey during the COVID-19 pandemic may have influenced participants’ attitudes. Despite these limitations, the use of standardized methodologies and consistent data collection instruments strengthens the validity of our findings and enables robust comparisons across age groups.

In conclusion, this study highlights the complexity of attitudes towards healthy eating among individuals in a disadvantaged region of Hungary, with a particular focus on older adults. Our findings emphasize the importance of targeted, tailored interventions that address the physical, mental, and socioeconomic barriers to adopting healthier diet. As Hungary develops its National Healthy Aging Program, this research provides a crucial basis for designing evidence-based strategies to promote dietary changes, reduce chronic disease risks, and support healthier aging across vulnerable populations.

## Data Availability

The data are available from the corresponding author upon reasonable request due to privacy or ethical concerns.
